# AP-1-Targeting Anti-Inflammatory Activity of the Methanolic Extract of *Persicaria chinensis*

**DOI:** 10.1155/2015/608126

**Published:** 2015-03-23

**Authors:** Muhammad Jahangir Hossen, Seung Cheol Kim, Young-Jin Son, Kwang-Soo Baek, Eunji Kim, Woo Seok Yang, Deok Jeong, Jae Gwang Park, Han Gyung Kim, Woo-Jae Chung, Keejung Yoon, Chongsuk Ryou, Sang Yeol Lee, Jong-Hoon Kim, Jae Youl Cho

**Affiliations:** ^1^Department of Genetic Engineering, Sungkyunkwan University, Suwon 440-746, Republic of Korea; ^2^Department of Animal Science, Patuakhali Science and Technology University, Patuakhali 8602, Bangladesh; ^3^Division of Gynecologic Oncology, Department of Obstetrics and Gynecology, Ewha Womans University Mokdong Hospital College of Medicine, Ewha Womans University, Seoul 158-710, Republic of Korea; ^4^Department of Pharmacy, Sunchon National University, Suncheon 540-742, Republic of Korea; ^5^Department of Pharmacy, College of Pharmacy and Institute of Pharmaceutical Science and Technology, Hanyang University, Ansan 426-791, Republic of Korea; ^6^Department of Life Science, Gachon University, Sungnam 461-701, Republic of Korea; ^7^Department of Veterinary Physiology, College of Veterinary Medicine, Biosafety Research Institute, Chonbuk National University, Jeonju 561-756, Republic of Korea

## Abstract

In traditional Chinese medicine, *Persicaria chinensis* L. has been prescribed to cure numerous inflammatory disorders. We previously analyzed the bioactivity of the methanol extract of this plant (Pc-ME) against LPS-induced NO and PGE_2_ in RAW264.7 macrophages and found that it prevented HCl/EtOH-induced gastric ulcers in mice. The purpose of the current study was to explore the molecular mechanism by which Pc-ME inhibits activator protein- (AP-) 1 activation pathway and mediates its hepatoprotective activity. To investigate the putative therapeutic properties of Pc-ME against AP-1-mediated inflammation and hepatotoxicity, lipopolysaccharide- (LPS-) stimulated RAW264.7 and U937 cells, a monocyte-like human cell line, and an LPS/D-galactosamine- (D-GalN-) induced acute hepatitis mouse model were employed. The expression of LPS-induced proinflammatory cytokines including interleukin- (IL-) 1*β*, IL-6, and tumor necrosis factor-*α* (TNF-*α*) was significantly diminished by Pc-ME. Moreover, Pc-ME reduced AP-1 activation and mitogen-activated protein kinase (MAPK) phosphorylation in both LPS-stimulated RAW264.7 cells and differentiated U937 cells. Additionally, we highlighted the hepatoprotective and curative effects of Pc-ME pretreated orally in a mouse model of LPS/D-GalN-intoxicated acute liver injury by demonstrating the significant reduction in elevated serum AST and ALT levels and histological damage. Therefore, these results strongly suggest that Pc-ME could function as an antihepatitis remedy suppressing MAPK/AP-1-mediated inflammatory events.

## 1. Introduction

Inflammation and innate immune response are considered beneficial for host survival [1] and are part of the complex biological response of living organisms to harmful stimuli, such as infection, cellular damage, and tissue injury [2]. Numerous cellular and biochemical alterations including downregulation of anti-inflammatory proteins and upregulation of proinflammatory gene products occur during inflammatory conditions to facilitate immune cell recruitment and to boost body's defensive mechanism [3, 4]. Nevertheless, the instability of immune homeostasis and prolonged inflammatory response can result in the development of various chronic diseases such as autoimmune disorders, cancer, and vascular diseases [5, 6]. Lipopolysaccharide (LPS) stimulates several proinflammatory mediator cytokines such as interferon, interleukin-1*β* (IL-1*β*), interleukin-6 (IL-6), and tumor necrosis factor-*α* (TNF-*α*) [1, 7, 8].

During LPS-induced inflammation, LPS binds to toll-like receptor 4 (TLR4) and stimulates the recruitment of both cytoplasmic MyD88 and TRIF adaptor proteins, which activate mitogen-activated protein kinase (MAPK) signaling [9]. The MAPK family consists of extracellular signal-regulated kinase (ERK), c-Jun N-terminal kinase (JNK), and p38. Continual activation of the MAPK signaling pathway has been shown to increase the activation of activator protein- (AP-) 1, a heterodimeric transcription factor, composed of c-Fos, c-Jun, ATF, and JDP families [10]. Activated AP-1 eventually upregulates the transcription of inflammatory genes containing the 12-O-tetradecanoylphorbol-13-acetate (TPA) DNA response element (TRE, 5′-TGAG/CTCA-3′) [11]. Indeed, it is known that the development of numerous human inflammatory diseases is accompanied with the activation of AP-1 [12]. Hence, targeting MAPK/AP-1 pathways is an attractive anti-inflammatory therapeutic approach.

Hepatitis, a liver disease associated with severe hepatocyte damage, is highly associated with acute or chronic inflammation caused by other infections, alcohol ingestion, certain medications, some industrial organic solvents and plants, and autoimmune diseases [13, 14]. Liver inflammation recruits numerous types of immune cells such as natural killer cells, T cells, dendritic cells, and macrophages [13]. Like other inflammatory diseases, the hepatic macrophages display prime pathophysiological roles in inducing liver injury by enormous production of reactive oxygen/nitrogen species and proinflammatory cytokines such as TNF-*α*, IL-1*β*, and IL-6 [13]. LPS/D-galactosamine- (D-GalN-) induced hepatitis in mice is a classical experimental model of severe liver injury involving the production of inflammatory cytokines and recruitment of inflammatory cells, leading to liver damage and dysfunction [15, 16].

*Persicaria chinensis *L. (Polygonaceae) is one of the representative medicinal plants that are widely used in tropical and subtropical Eastern Asia [17]. The Malaysian Chinese community and Tamang community of Nepal have been known to prescribe this plant to treat various lung diseases [18, 19]. Malaysian communities and Indian tribes have used the methanolic extract of leaves to cope with infectious diseases and ulcers [17, 20]. In addition, numerous previous studies have reported the importance of* P. chinensis* as an anti-inflammatory plant [18–20], but the molecular ethnopharmacological evidence is still ambiguous. Recently, we demonstrated that 95% methanol extract of the aerial parts of this plant (Pc-ME) can effectively ameliorate inflammatory responses in HCl/EtOH-induced gastritis and TLR4-activated macrophages through the suppression of Syk/Src/NF-*κ*B [17]. Previously, it was stated that MAPKs play an important role in the regulation of LPS-induced inflammation by controlling AP-1 activation [21] and are strongly linked to the induction of hepatitis [22, 23]. This prompted us to further examine the molecular inhibitory effects of Pc-ME on the AP-1 pathway and we assumed that this extract may be capable of attenuating hepatitis symptoms. To conquer our hypothesis, we used lipopolysaccharide- (LPS-) treated macrophages RAW264.7 cell line and human pleura/pleural effusion monocyte-like cell line U937 and LPS/D-GalN-induced hepatitis mouse model. Holistic molecular approaches including reporter gene assays, immunoprecipitation analysis, and histopathological and hematopathological investigation were also used to confirm our assumption.

## 2. Materials and Methods

### 2.1. Materials

Quercetin, 3-(4,5-dimethylthiazol-2-yl)-2,5-diphenyltetrazolium bromide (MTT), and lipopolysaccharide (LPS,* E. coli* 0111:B4) were purchased from Sigma Chemical Co. (St. Louis, MO, USA). Luciferase constructs containing promoters for AP-1 were a gift from Professor Chung, Hae Young (Pusan National University, Pusan, Korea). Fetal bovine serum (FBS) and RPMI1640 were obtained from Gibco (Grand Island, NY, USA). RAW264.7 cells, a BALB/c-derived murine macrophage cell line (number TIB-71); U937 cells, a human pleura/pleural effusion monocyte-like cell line (number CRL-1593.2); and HEK293 cells, a human embryonic kidney cell line (number CRL-1573), were purchased from American Tissue Culture Center (Rockville, MD, USA). Luciferase constructs containing binding sites for AP-1 were used as reported previously [24, 25]. All other chemicals were obtained from Sigma. Phosphospecific or total antibodies to lamin A/C, c-Fos, c-Jun, ERK, JNK, p38, MEK1/2, MKK4, and *β*-actin used in this study were purchased from Cell Signaling Technology (Beverly, MA, USA).

### 2.2. Pc-ME Preparation

Pc-ME (Code number: PBID 110601) was obtained from the Plant Extract Bank in the Plant Diversity Research Center (Daejeon, Korea; http://extract.kribb.re.kr/extract/f.htm, e-mail: mplantext@kribb.re.kr), as reported previously [17].

### 2.3. Drug Treatment

In case of cellular experiments, Pc-ME prepared in 100% DMSO at a concentration of 100 mg/mL was diluted with culture medium. For animal experiment, Pc-ME (200 mg/kg) was resuspended in 1% sodium carboxymethylcellulose (CMC), as reported previously [26], and LPS (10 *μ*g/kg)/D-GalN (1 g/kg) was dissolved in phosphate-buffered saline (PBS).

### 2.4. *In Vitro* Studies

#### 2.4.1. Cell Culture

The cancerous macrophage line RAW264.7 and human pleura/pleural effusion monocyte-like cell line U937 were maintained in RPMI1640, while human embryonic kidney cell line HEK293 was cultured in DMEM medium, each supplemented with 10% heat-inactivated FBS, glutamine, and penicillin/streptomycin at 37°C during 5% CO_2_. Before Pc-ME treatment, U937 cells were treated with PMA (20 nM) for 12 h.

#### 2.4.2. Cell Viability Test

After preincubation of RAW264.7, HEK 293, and U937 cells (1 × 10^6^ cells/mL) for 18 h, Pc-ME (0, 100, 200, and 300 *μ*g/mL) was added to the cell suspensions and incubated for 24 h. The effect of Pc-ME on cell viability was tested by a conventional MTT assay, according to previous reports [27, 28]. In brief, at 3 h prior to culture termination, 10 *μ*L of MTT solutions (10 mg/mL in phosphate-buffered saline, pH 7.4) was added and cells were continuously cultured until assay termination. The incubation was halted by the addition of 15% sodium dodecyl sulphate to each well to solubilize the formazan and absorbance at 570–630 nm (OD_570–630_) was measured using a Spectramax 250 microplate reader.

#### 2.4.3. mRNA Analysis by Semiquantitative Reverse Transcriptase-Polymerase Chain Reaction (RT-PCR) and Real-Time PCR

To determine mRNA expression levels of proinflammatory cytokine genes, RAW264.7 or U937 cells were exposed to Pc-ME (0, 100, and 300 *μ*g/mL) for 30 min (RAW264.7 cells) or 3 h (U937 cells) before incubation with LPS (1 *μ*g/mL for RAW264.7 cells and 10 *μ*g/mL for U937 cells) for 6 h (RAW264.7 cells) or 12 h (U937 cells). Total RNA was prepared with TRIzol reagent (Gibco) according to the manufacturer's instructions and stored at −70°C for later use. Semiquantitative RT-PCR and real-time PCR reactions were also carried out, according to previous report [29]. The primers (Bioneer, Seoul, Korea) used in this study are listed in Table 1.

#### 2.4.4. Plasmid Transfection and Luciferase Reporter Gene Activity Assay

HEK293 cells (1 × 10^6^ cells/mL in 12-well plates) were transfected with plasmids (*β*-galactosidase and AP-1-Luc) under cotransfection with an inducing molecule (MyD88, TRIF, or PMA) using the polyethyleneimine (PEI) method. The cells were treated with Pc-ME (0, 100, 200, and 300 *μ*g/mL) or quercetin (0, 20, 40, and 80 *μ*M) for 12 h until harvesting. Luciferase activity was determined by the Luciferase Assay System (Promega, Madison, WI, USA), as previously reported [30, 31].

### 2.5. *In Vivo* Studies

#### 2.5.1. Animals

Male C57BL/6 mice (6–8 weeks old, 17–21 g) were purchased from DAEHAN BIOLINK (Chungbuk, Korea) and were housed in groups of 6–8 mice under a 12 h light/dark cycle (lights on at 6 a.m.). Water and pellet diets (Samyang, Daejeon, Korea) were supplied* ad libitum*. Animals were cared for in accordance with the guidelines issued by the National Institute of Health for the Care and Use of Laboratory Animals (NIH Publication 80-23, revised in 1996). Studies were performed in accordance with guidelines established by the Institutional Animal Care and Use Committee at Sungkyunkwan University (Suwon, Korea; approval ID: SKKUBBI 12-6-1).

#### 2.5.2. LPS/D-GalN-Induced Hepatitis Mouse Model

A model of experimental liver inflammation was induced by LPS injection according to a previously published method [32]. Briefly, five-week-old C57BL/6 mice were treated orally with Pc-ME (200 mg/kg) once a day for six days with the aid of crop needles. One hour after the final administration of Pc-ME, LPS (10 *μ*g/kg) and D-GalN (1 g/kg) were injected intraperitoneally. Each animal was anesthetized with an overdose of urethane 1 hour after administration of hepatitis inducers, and blood was collected by cardiac puncture. The livers were then excised and gently rinsed with PBS. Serum was obtained by centrifugation of blood at 3,000 rpm for 15 min. The levels of serum alanine aminotransferase (ALT) and aspartate aminotransferase (AST) were measured with a Roche Modular spectrophotometric autoanalyzer.

#### 2.5.3. Histopathology

The histopathological observation was also performed as previously described [33]. Briefly, tissue samples taken from the liver of the mice at 8 h after challenge with LPS and D-GalN were fixed with 10% formalin in PBS and then embedded in paraffin. Approximately 4 *μ*m thin tissue sections were stained with hematoxylin and eosin for histopathological examination.

### 2.6. Preparation of Total Lysates, Nuclear Extracts, and Immunoblotting

*In vivo* samples (liver tissues from mice treated with Pc-ME (0 and 200 mg/kg)) or* in vitro* samples (RAW264.7 cells (5 × 10^6^ cells/mL) stimulated with LPS for various time points (2, 3, 5, 15, 30, and 60 min) in the presence or absence of Pc-ME (0 to 300 *μ*g/mL) or PMA-treated U937 cells stimulated with LPS for 30 and 60 min during Pc-ME (0 and 300 *μ*g/mL) exposure) were washed three times in cold PBS with 1 mM sodium orthovanadate and lysed by a sonicator or a Tissuemizer in lysis buffer (20 mM Tris-HCl, pH 7.4, 2 mM EDTA, 2 mM ethyleneglycotetraacetic acid, 50 mM *β*-glycerophosphate, 1 mM sodium orthovanadate, 1 mM dithiothreitol, 1% Triton X-100, 10% glycerol, 10 *μ*g/mL aprotinin, 10 *μ*g/mL pepstatin, 1 mM benzamide, and 2 mM PMSF) for 30 min with rotation at 4°C. The lysates were clarified by centrifugation at 16,000 ×g for 10 min at 4°C and stored at −20°C until needed.

Nuclear extracts were prepared in a three-step procedure with RAW264.7 cells stimulated with LPS for 15, 30, 60, and 120 min in the presence or absence of Pc-ME (0 and 300 *μ*g/mL), as reported previously [34]. The cells were collected with a rubber policeman, washed with 1 × PBS, and lysed in 500 *μ*L lysis buffer containing 50 mM KCl, 0.5% Nonidet P-40, 25 mM HEPES (pH 7.8), 1 mM phenylmethylsulfonyl fluoride, 10 *μ*g/mL leupeptin, 20 *μ*g/mL aprotinin, and 100 *μ*M 1,4-dithiothreitol (DTT) on ice for 4 min. Cell lysates were then centrifuged at 19,326 ×g for 1 min in a microcentrifuge. In the second step, the pellet (the nuclear fraction) was washed once in washing buffer, which was the same as the lysis buffer but without Nonidet P-40. In the final step, nuclei were treated with an extraction buffer (lysis buffer containing 500 mM KCl and 10% glycerol). The nuclei/extraction buffer mixture was frozen at −80°C and then thawed on ice and centrifuged at 19,326 ×g for 5 min. The supernatant was collected as a nuclear extract.

Soluble cell lysates or the nuclear extracts were immunoblotted and total or phosphorylated protein levels of transcription factors (lamin A/C, c-Fos, and c-Jun), ERK, JNK, p38, MEK1/2, MKK4, and *β*-actin (as a control) were visualized, according to a previously published method [35].

### 2.7. Statistical Analysis

**Figure 1:**
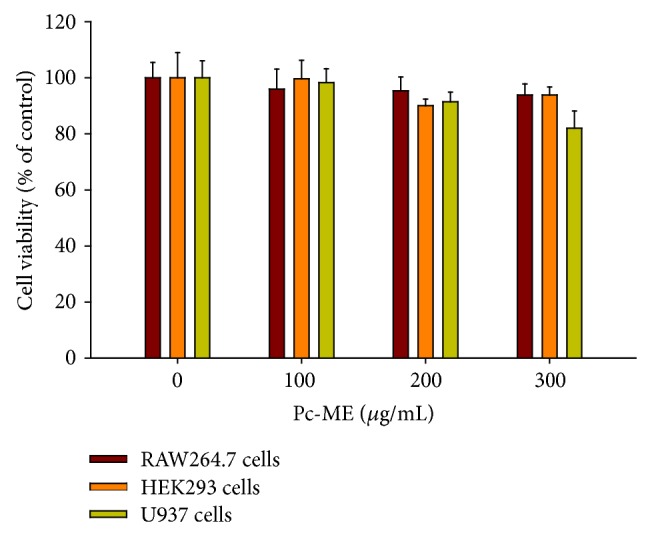
Cell viability of RAW264.7, HEK293, and U937 cells was determined using the MTT assay.

**Figure 2:**
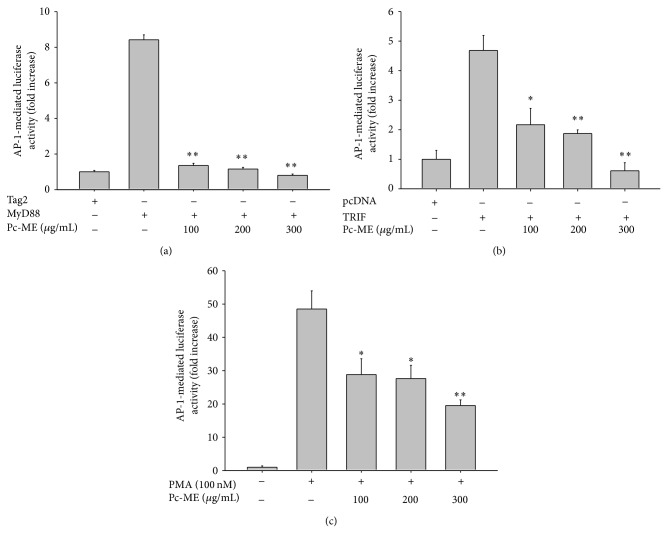
Effect of Pc-ME on the reporter gene assay. The promoter binding activity of the transcription factor AP-1 was analyzed using a reporter gene assay in HEK293 cells transfected with plasmid constructs AP-1-Luc (1 *μ*g/mL) or *β*-gal (as a transfection control) with 1 *μ*g/mL of MyD88 (a) or TRIF (b) and 100 nM PMA (c) in the presence of Pc-ME. Luciferase activity was measured using a luminometer. ^**^ *P* < 0.01 compared with control.

**Figure 3:**
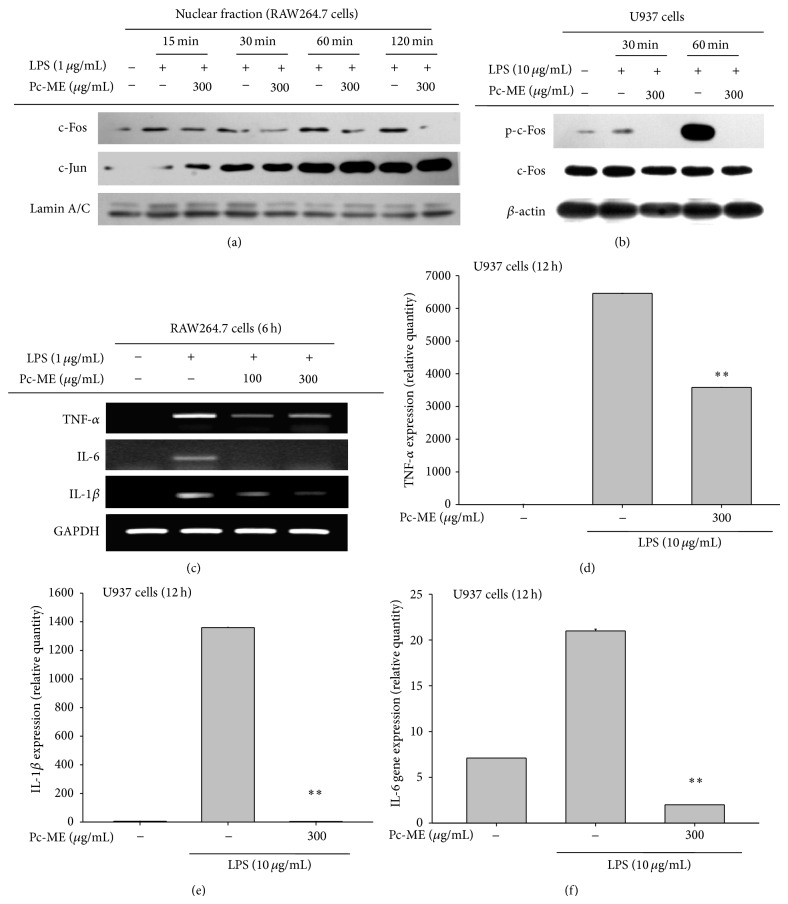
Effect of Pc-ME on the activation of proinflammatory cytokines and transcriptional regulation. (a) Levels of c-Fos, c-Jun, and lamin A/C in nuclear fractions were determined by immunoblot analysis in RAW264.7 cells. (b) Phospho- or total protein levels of c-Fos and *β*-actin in cell lysates were determined by immunoblot analysis in U937 cells. (c) mRNA levels of TNF-*α*, IL-1*β*, IL-6, and GAPDH were determined by semiquantitative RT-PCR in RAW264.7 cells. (d–f) mRNA levels of TNF-*α*, IL-1*β*, and IL-6 were determined by real-time RT-PCR in U937 cells.

**Figure 4:**
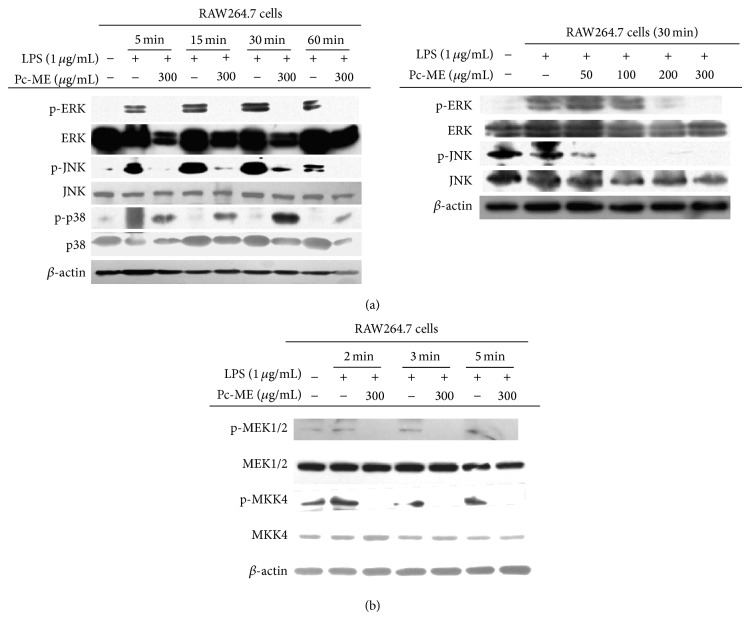
Effect of Pc-ME on the activation of upstream signaling enzymes of AP-1 translocation. (a and b) Phospho- and total protein levels of ERK, JNK, p38, MEK1/2, MKK4, and *β*-actin in cell lysates were determined by immunoblotting analysis.

**Figure 5:**
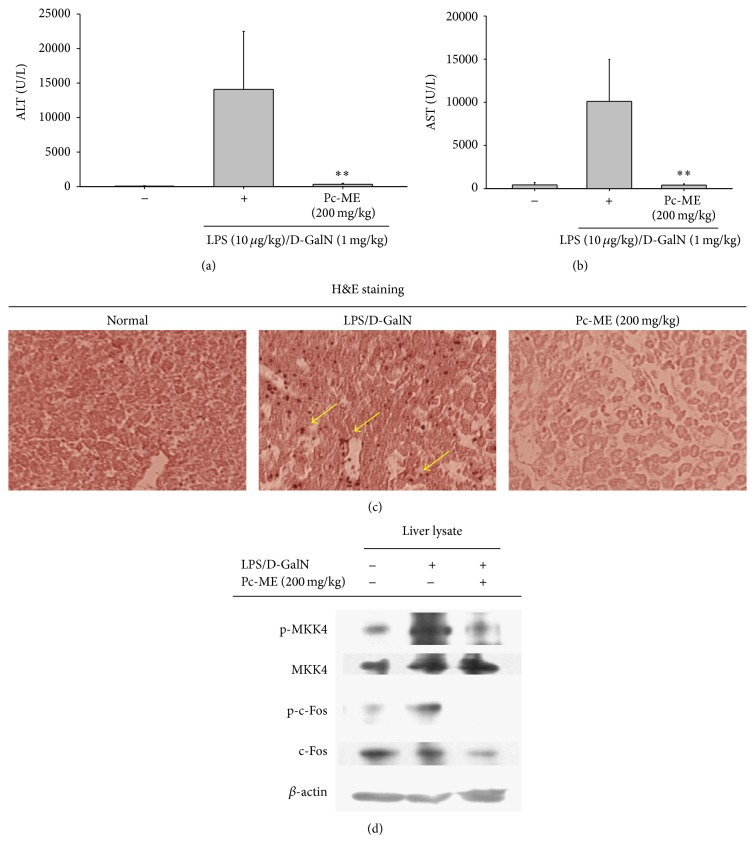
Effect of Pc-ME on LPS/D-GalN-induced hepatitis in mice. Mice were orally treated with Pc-ME (200 mg/kg) for six days before intraperitoneal injection of LPS/D-GalN. After 1 h, mice were sacrificed to collect blood samples and liver sections for biochemical parameter analysis of (a) ALT, (b) AST, and (c) histopathological examination. (d) Phospho- or total protein levels of MKK4, c-Fos, and *β*-actin in liver lysates were determined by immunoblot analysis.

**Figure 6:**
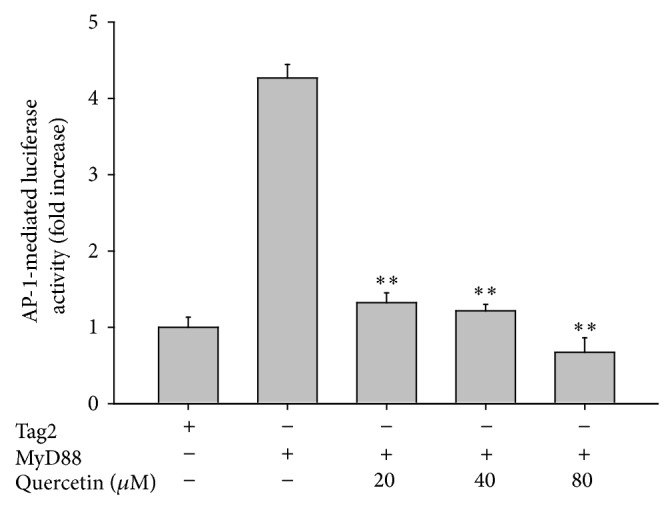
Effect of quercetin on the activation of upstream signaling enzymes of AP-1 in a reporter gene assay. The promoter binding activity of the transcription factor AP-1 was analyzed using a reporter gene assay in HEK293 cells transfected with plasmid constructs AP-1-Luc (1 *μ*g/mL) and *β*-gal (as a transfection control) with 1 *μ*g/mL of MyD88 in the presence of quercetin. Luciferase activity was measured using a luminometer. ^**^ *P* < 0.01 compared with control.

All data are expressed as the mean ± standard deviation (SD) of an experiment performed with six (Figures 1, 2, and 6) or three (Figures 3, 4, and 5) samples for* in vitro* test and six mice of each group for* in vivo* tests (Figure 5). Statistical comparisons were carried out by ANOVA/Scheffe's post hoc test and Kruskal-Wallis/Mann-Whitney tests. A *P* value <0.05 was considered statistically significant. All statistical tests were performed with the computer program SPSS 17 for Windows XP. Similar results were found in an additional independent set of* in vitro* and* in vivo* experiments performed under the same conditions.

## 3. Results

### 3.1. Effect of Pc-ME on Cell Viability

As shown in Figure 1, the viability of RAW264.7, HEK293, and U937 cells was not significantly affected by treatment with Pc-ME up to 300 *μ*g/mL compared with that of the cells receiving no LPS treatment.

### 3.2. Effect of Pc-ME on the Transcriptional Activation of AP-1

We next performed a transfection experiment with the AP-1-Luc construct and HEK293 cells and used luciferase assays to examine whether Pc-ME suppressed the functional activation of AP-1. We found that AP-1-mediated luciferase activity was increased by PMA treatment (up to 50-fold) or cotransfection with adaptor molecules TRIF (up to 4.5-fold) and MyD88 (up to 8-fold), whereas Pc-ME treatment significantly (*P* < 0.01) and dose-dependently (100, 200, and 300 *μ*g/mL) inhibited this upregulation (Figure 2), suggesting that AP-1 activation is a major pharmacological target of Pc-ME.

AP-1 transcription factor is known to have a major regulatory role in inflammatory gene expression, so we examined the suppressive effect of Pc-Me on the activation and translocation of AP-1 after treatment with Pc-ME.  Figure 3(a) shows the increase in nuclear level of the AP-1 c-Fos subunit due to time-dependent inhibition by Pc-ME (15, 30, 60, and 120 min). Similar time-dependent (30 and 60 min) inhibitory patterns of c-Fos expression were confirmed by whole lysate extraction from U937 cells (Figure 3(b)).

### 3.3. Effects of Pc-ME on LPS-Induced Proinflammatory Cytokine Production

Lee et al. [33] and Feldmann [36] have suggested that TNF-*α*, IL-1*β*, and IL-6 are crucial mediators of the development of inflammatory diseases. We further investigated the effect of Pc-ME on proinflammatory gene expression in RAW264.7 cells and U937 cells after LPS treatment. RT-PCR results demonstrated a significant concentration-dependent decrease in LPS-induced upregulation of TNF-*α*, IL-1*β*, and IL-6 mRNA levels in Pc-ME-treated RAW264.7 cells (Figure 3(c)). In parallel, real-time PCR (Figures 3(d) to 3(f)) in U937 cells clearly showed that LPS was able to induce the upregulation of proinflammatory cytokines such as TNF-*α* up to 6,460-fold, IL-1*β* up to 1,360-fold, and IL-6 up to 20-fold, whereas Pc-ME (300 *μ*g/mL) strongly (*P* < 0.01) inhibited this.

### 3.4. Effect of Pc-ME on Upstream Signaling  for AP-1 Activation

It has been reported [37] that phosphorylation of MAPK (ERK, JNK, and p38) plays a pivotal role in the regulation of LPS-induced inflammatory mediators, so we performed Western blot analysis to determine the inhibitory activity of Pc-ME on proinflammatory mediators. LPS significantly elevated the phosphorylation of ERK, JNK, and p38, whereas Pc-ME pretreatment strongly and time-dependently (5, 15, 30, and 60 min) suppressed LPS-induced phosphorylation of JNK and ERK but not that of p38 (Figure 4(a), left panel) in RAW264.7 cells. The right panel in Figure 4(a) shows that Pc-ME (50, 100, 200, and 300 *μ*g/mL) dose-dependently blocked the phosphorylation of ERK and JNK, which validates our experimental findings and confirms our expectations. Upstream signaling enzymes (MEK1/2 and MKK4) contributing to ERK and JNK phosphorylation were appreciably blocked by Pc-ME at 2, 3, and 5 min of LPS treatment (Figure 4(b)), which demonstrates that MEK1/2 and MKK4 might be targeted by Pc-ME in its AP-1-suppressive anti-inflammatory actions in LPS-stimulated RAW264.7 cells. In concurrence with our LPS/D-GalN-treated mice hepatitis experimental model, the upregulation of phosphorylated MKK4 and c-Fos in the liver tissue was markedly suppressed by orally administered Pc-ME (Figure 5(d)).

### 3.5. Hepatoprotective Effect of Pc-ME on LPS/D-GalN-Induced Liver Injury in Mice

We used a mouse model of LPS/D-GalN-induced liver injury to investigate the* in vivo* hepatoprotective effect of Pc-ME. LPS/D-GalN-triggered ALT (14,000 U/L) and AST (10,000 U/L) protein levels were significantly (*P* < 0.01) decreased by Pc-ME (Figures 5(a) and 5(b)). Moreover, histopathological analysis demonstrated that the liver sections of the LPS/D-GalN group displayed more neutrophil recruitment, as assessed by bigger sized and increased numbers dark spots (see arrows in Figure 5(c)), compared with the saline-treated control groups; in contrast, the Pc-ME-treated groups exhibited lower neutrophil numbers (Figure 5(c)), which demonstrates the strong hepatoprotective activity of Pc-ME.

### 3.6. Effect of Quercetin on AP-1 Activity

Cotransfection with the adaptor molecule MyD88 enhanced AP-1-mediated luciferase activity by 4.5-fold; quercetin, a major flavonoid from Pc-ME [17], significantly (*P* < 0.01) and dose-dependently inhibited this upregulation (Figure 6), which demonstrates that AP-1 activation is a major pharmacological target of Pc-ME and its ingredient quercetin.

## 4. Discussion

While* P. chinensis* has high ethnopharmacological worth in Eastern Asian countries, the molecular mechanisms underlying its anti-inflammatory activity are still unknown. Recently, our studies have revealed that* P. chinensis* methanol extract exhibits strong antigastritis activity and is able to block NF-*κ*B activation via suppression of Src and Syk activities [17]. However, Src/Syk-linked activation of NF-*κ*B is not the only important regulatory loop of inflammatory reaction. In addition, it has been reported that AP-1 activated by MAPK plays another crucial roles in inflammatory reaction [11, 38, 39]. In the present study, therefore, we aimed to elucidate inhibitory mechanism of Pc-ME on AP-1 function* in vitro* and* in vivo* by using LPS-activated macrophages and LPS/D-GalN-triggered hepatitis model.

It has been shown that reporter gene luciferase assay performed in conjunction with HEK293 cells transfected with Luc constructs and adaptor molecules, essential for TLR signaling [40], is a reasonable approach for studying functional activation of transcription factors [41, 42]. Therefore, to examine the ability of Pc-ME to suppress AP-1 function, we first employed the luciferase assay using HEK293 cells transfected with the AP-1-Luc construct. As expected, AP-1-mediated luciferase activity was enhanced up to 4.5- to 48.5-fold by PMA treatment or cotransfection with adaptor molecules (TRIF and MyD88), and Pc-ME notably inhibited this upregulation in a dose-dependent manner (Figure 2). Moreover, nuclear translocation of c-Fos was reduced by Pc-ME treatment in a time-dependent manner (Figure 3(a)), implying that AP-1 family of transcription factors can be functionally inactivated and that their upstream kinases responsible for AP-1 phosphorylation can be targeted.

Several earlier reports have suggested that several proinflammatory cytokines such as TNF-*α*, IL-1*β*, and IL-6 play an important role in boosting proinflammatory roles of macrophages [43–45]. We therefore further tested whether these proinflammatory cytokines can be also suppressed by Pc-ME using LPS-treated RAW264.7 cells. The mRNA analysis of these cytokines by RT-PCR in RAW264.7 cells (Figure 3(c)) and by real-time PCR in U937 cells (Figures 3(d)–3(f)) revealed that mRNA levels of TNF-*α*, IL-1*β*, and IL-6 were strongly upregulated by LPS treatment, while Pc-ME significantly and dose-dependently (100 and 300 *μ*g/mL) inhibited such upregulation, indicating that AP-1 suppression by Pc-ME may be associated with blockade of these proinflammatory cytokines as well as its suppressive activity on the expression of iNOS and COX-2 [17]. In fact, a number of studies have also reported that many known herbal medicines such as* Polygonum hydropiper*,* Pistacia integerrima*,* Phaseolus angularis*,* Morus bombycis* Koidzumi, and* Sanguisorba officinalis* possess AP-1 pathway inhibitory activity as their pharmacological target [26, 46–49]. Therefore, the fact that Pc-ME is able to inhibit AP-1 pathway could be also accepted as a general anti-inflammatory mechanism of this plant.

As MAPKs play a vital role in the regulation of LPS-induced inflammation by controlling AP-1 activation [21], we examine the molecular inhibitory effects of Pc-ME on the AP-1 pathway. Toward this goal, we analyzed the inhibitory effect of Pc-ME on MAPKs and their upstream signaling enzymes [50]. The results of our study demonstrated that Pc-ME treatment time-dependently (5, 15, 30, and 60 min) blocked ERK and JNK phosphorylation (Figure 4(a) left panel), potentially leading to significant attenuation of AP-1 activation in response to LPS. The dose-dependent (50 to 300 *μ*g/mL) inhibition pattern of the same MAPK phosphorylation by this extract (Figure 4(a) right panel) strongly supported our experimental condition and hypothesis. The phosphorylation of MEK1/2 and MKK4, the upstream enzymes of ERK and JNK, respectively, was also strikingly suppressed by Pc-ME in LPS challenges of 2, 3, and 5 min (Figure 4(b)), confirming the MAPK inhibitory activity of this extract.

MAPK activation and subsequent AP-1 activation are strongly linked to the induction of hepatitis [22, 23]. So, we guess that AP-1-inhibitory extract, Pc-ME, is capable of attenuating hepatitis symptoms. To test this hypothesis, we employed LPS/D-GalN-intoxicated acute liver injury model, which is a classical system for screening hepatoprotective remedy* in vivo* [32]. Our previous study indicating that Pc-ME can protect against HCl/EtOH-induced gastritis by inhibiting Src/Syk of NF-*κ*B [17] has demonstrated the oral effectiveness of this extract. In the present study, expectedly, Pc-ME treatment (200 mg/kg) effectively ameliorated the LPS/D-GalN-induced liver damage (Figure 5(c)), including reversion of LPS/D-GalN-elevated hepatic ALT (Figure 5(a)) and AST (Figure 5(b)) enzyme levels. In the* in vivo* hepatic tissue, the upregulated phosphorylation of MKK4 and c-Fos (component of AP-1) was also noticeably blocked by Pc-ME treatment (Figure 5(d)), which strongly authenticated our findings. In addition, quercetin, a major antioxidative and anti-inflammatory compound of* P. chinensis* [17], also drastically diminished the AP-1 activation in a dose-dependent pattern (Figure 6), supporting that MAPK/AP-1-targeted anti-inflammatory activity of Pc-ME could be quercetin-derived anti-inflammatory action. Although numerous numbers of medicinal plants or edible fruits such as* Panax ginseng*,* Fagonia schweinfurthii*, fermented soybean,* Davilla elliptica*, and* Boesenbergia rotunda* have been reported to show antihepatitis activities [51–55], only few plants are now clinically prescribed. It was revealed that Pc-ME is able to strongly suppress both NF-*κ*B [17] and AP-1 activity; we will further validate whether Pc-ME can be also clinically developed as a new antihepatitis herbal drug.

**Figure 7:**
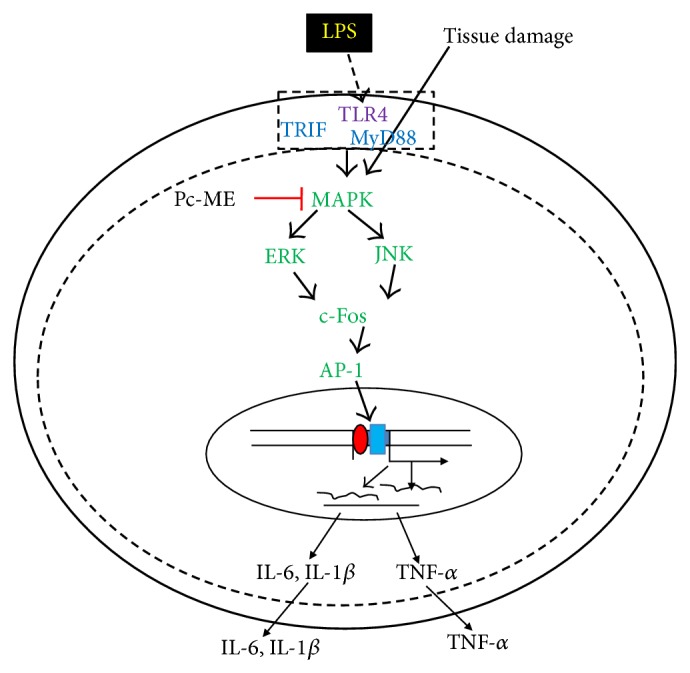
Putative mechanism of Pc-ME-mediated anti-inflammatory responses.

In summary, our* in vivo* and* in vitro* assays demonstrated that Pc-ME significantly reduced the levels of LPS-mediated proinflammatory cytokines (TNF-*α*, IL-1*β*, and IL-6), and that MAPK/AP-1 inactivation by this extract contributes to these inhibitory effects as summarized in Figure 7. The strong antihepatotoxic activity of Pc-ME* in vivo* was observed in a mouse model of LPS/D-GalN-induced liver injury, indicating that Pc-ME could potentially be used as a hepatoprotective remedy.

## Figures and Tables

**Table 1a:** (a) RT-PCR primers

Name	Sequence (5′ to 3′)	
TNF-*α*	F	TTGACCTCAGCGCTGAGTTG
	R	CCTGTAGCCCACGTCGTAGC

IL-6	F	GGAAATCGTGGAAATGAG
	R	GCTTAGGCATAACGCACT

IL-1*β*	F	CAGGATGAGGACATGAGCAC
	R	CTCTGCAGACTCAAACTCCA

GAPDH	F	CAA TGA ATA CGG CTA CAG CAA C
	R	AGG GAG ATG CTC AGT GTT GG

**Table 1b:** (b) Real-time PCR primers

Name	Sequence (5′ to 3′)	
TNF-*α*	F	GAAAGCATGATCCGGGACGTG
	R	GATGGCAGAGAGGAGGTTGAC

IL-6	F	AAGCCAGAGCTGTGCAGATGAGTA
	R	CTTGGTCACCGACGTCCTGT

IL-1*β*	F	CCGACCACCACTACAGCAAG
	R	GGGCAGGGAACCAGCATCTT

GAPDH	F	TGGAAGGACTCATGACCACA
	R	AGGGGTCTACATGGCAACTG
